# HEV Infection in the Context of Prior HBV-Related Liver Injury: Case Series

**DOI:** 10.3390/idr16050070

**Published:** 2024-09-06

**Authors:** Mihaela-Cristina Olariu, Mihai-Cezar Filipescu, Andreea Marilena Pauna, Madalina Simoiu, Alina Maria Borcan

**Affiliations:** 1 Department of Infectious Diseases, University of Medicine and Pharmacy “Carol Davila”, 020021 Bucharest, Romania; mihaela.olariu@umfcd.ro; 2 National Institute for Infectious Diseases “Matei Balș”, 021105 Bucharest, Romania; mihai-cezar.filipescu@rez.umfcd.ro (M.-C.F.); borcanalinamaria@yahoo.com (A.M.B.); 3 Department of Epidemiology I, University of Medicine and Pharmacy “Carol Davila”, 020021 Bucharest, Romania; 4 Military Medical Institute, 010919 Bucharest, Romania; 5 Department of Parasitology, University of Medicine and Pharmacy “Carol Davila”, 020021 Bucharest, Romania; 6 Department of Microbiology, University of Medicine and Pharmacy “Carol Davila”, 020021 Bucharest, Romania

**Keywords:** hepatitis, superinfection, HEV, HBV, co-infection

## Abstract

Hepatitis E virus (HEV) is a common cause of acute hepatitis, with increasing incidence in Europe, including Romania. Concurrently, Romania has a high prevalence of chronic hepatitis B (CHB). There is limited research on the clinical presentation and outcomes of HEV infection in patients with pre-existing chronic hepatitis B (CHB), especially in resource-rich settings. Most literature data come from South, East, and Southeast Asia. A review of the literature on HEV and HBV co-infection indicates a severe prognosis, particularly in patients with underlying liver disease. However, the cases in this study, which did not display cirrhosis, showed varied outcomes. The role of anti-HBV treatment in improving prognosis remains uncertain and warrants further investigation. Acute HEV infection superimposed on chronic HBV infection poses significant clinical challenges, with outcomes ranging from full recovery to fatality. Preventive measures, including sanitation and vaccination against HBV, are crucial. More studies are needed to establish effective treatment protocols for this co-infection. In this study, we will analyze the clinical setting, diagnosis, particularities, and outcomes of five such cases of dual hepatotropic viral infection recorded over a period of 6 years (2018–2023) at a large Infectious Diseases clinic in Bucharest, Romania.

## 1. Introduction

Hepatitis E virus (HEV) is a common cause of acute hepatitis, especially in developing countries [1]. It is caused by a RNA virus without an envelope, from the family *Hepeviridae*. This virus has the ability to infect humans, mammals, birds, and fish [2]. A hepatotropic virus with a direct cytopathogenic effect, HEV is transmitted through a fecal–oral route. It is usually found in developing countries with poor sanitation. Hepatitis B virus (HBV), on the other hand, pertaining to the *Hepadnaviridae* family of hepatotropic virus, is a DNA virus, with the capability to cause chronic inflammation of the liver, cirrhosis, hepatic cancer, and ultimately death. An ubiquitous pathogen, its European prevalence roughly ranges from a low incidence [3] (in Northern and Western European countries) to an average prevalence (in Southern and Eastern Europe). New epidemiologic findings in Europe (including Romania) show a decrease in chronic hepatitis B (CHB) and an increase in incidence for acute hepatitis E. In Romania, in particular, in the case of chronic HBV infection, most recent data [4] show an HBsAg carrier rate of 4.3% in the general population, placing Romania among the European Union countries with an elevated prevalence of this infection. Regarding HEV acute hepatitis, data [5] from 2019 showed an IgG seroprevalence of upwards to 17.1%. On a continental level, it has been shown [6] that the proportion of autochthonous HEV infections has been steadily increasing since 2005.

Given its relatively rare incidence in Europe until recently and the fact that it is an under-recognized cause of acute hepatitis in high-income countries, relatively few studies have been conducted on the clinical presentation and outcome of HEV infection in patients already presenting with a chronic hepatitis B virus (HBV) infection. We will present a series of five such cases from a large Infectious Diseases clinic in Bucharest, Romania, admitted during a period of 6 years (2018–2023) in order to quantify if a more severe clinical outcome was apparent.

## 2. Case Series

**Case 1:** A 40 year-old male smoker, of normal weight, and a rural resident, was admitted in his third week of illness, consisting of headache, nausea, anorexia, myalgia, and jaundice. He was initially admitted to another clinic for 2 weeks, where blood works showed elevated liver enzymes (ALT approximately 100 times the upper limit of normal (ULN) and AST approximately 75 times the ULN), hyperbilirubinemia (total bilirubinemia approximately 14 times the ULN) and positive serologic markers for HBsAg. Computed tomography (CT) scans revealed hepatomegaly and medium-quantity ascites. Given the high suspicion for an infectious cause of the presentation, the patient was transferred into our clinic, where the clinical exam upon admittance revealed hepatalgia in the presence of an enlarged liver with an increased consistency, marked jaundice, acholic, stools and hyperchrome urine. Biologically, the patient had impaired coagulation tests (prothrombin concentration of 51%), persistently increased liver enzymes together with hyperbilirubinemia, and a viral serology (for an easier read, all serological testing and molecular testing is presented in Table 1) consistent with acute hepatitis due to both HBV and HEV (positive IgM HVEAb (antibodies) and IgM HBsAb), indicating possible co-infection. Treatment was mainly supportive, including the administration of vitamin K and corticotherapy. The outcome was favorable, with a slow resolution of the jaundice and normalization of liver enzymes.

**Case 2:** A 69-year-old patient, recently (6 months before admittance) diagnosed with small cell B-cell lymphoma (SCBCL), and at that point undergoing treatment with monoclonal antibodies (MABs), was admitted into our clinic for further investigations after a routine check-up found elevated liver enzymes (approximately 10 times the ULN). The clinical exam was within the normal parameters, noting a lack of jaundice but with an enlarged spleen and a normal-sized liver, albeit with a slightly higher consistency. Blood works revealed mild leukopenia and elevated liver enzymes (ALT 6 times the ULN) together with a serology consistent with acute HEV hepatitis (IgM anti-HEV antibodies) atop of chronic HBV hepatitis (positive HBsAg and HBc IgG Ab) associated with hepatitis D virus (HDV) (positive IgG HVD) infection together with a positive HBV viral load of 38,700,000 IU/mL. A diagnosis of acute HEV hepatitis and chronic HBV and HDV hepatitis (most likely reactivated in the context of iatrogenic immunosuppression due to MABs treatment) was therefore established. The patient received supportive treatment and antiviral therapy with Entecavir was initiated. The outcome was favorable, with HBsAg seroconversion after 4 months of antiviral therapy.

**Case 3:** A 38-year-old cachectic female, with a history of chronic hepatitis B spanning 8 years and without aethiologic treatment at the moment of admission, presented with intense jaundice, nausea, and emesis which manifested one month before presentation. Initially, she was evaluated at a surgical service where an obstructive cause of the jaundice was excluded and she was redirected to the Infectious Disease (ID) department. The clinical exam upon admission was quasi-normal, with the exception of jaundice. Blood works showed increased liver enzyme levels (ALAT 25 times the ULN) and hyperbilirubinemia (TB 3 times the ULN). Serologic tests were consistent with the diagnosis of acute VHE hepatitis (positive IgM HVEAb) and CHB in an immune-active phase (HBsAg positive, HBeAg positive, IgM HBcAb positive). Under supportive treatment, evolution was favorable, with the normalization of liver enzymes coupled with the remission of the jaundice.

**Case 4:** A 34-year-old male, previously diagnosed with chronic HBV infection, was admitted with jaundice, acholic stools, and hyperchromic urine along with flu-like symptoms (fever, chills, cough) which manifested 7 days prior. The clinical exam revealed jaundice and a moderately enlarged liver. Mild thrombocytopenia and abnormal coagulation tests (notable lower prothrombin levels) were found, along with increased liver enzyme levels, hyperbilirubinemia (total bilirubin 4 times the ULN), and a serology consistent with an acute HEV and chronic HBV infection (positive HBsAg, HBeAb, IgG HBc Ab, IgM HEVAb, HBV viral load 20,000,000 IU/mL). Treatment with Rifaximin, plasma transfusions, and supportive care was initiated. Under said treatment, the clinical course was marked by a severe and rapid deterioration and the patient was admitted in the ICU, where Entecavir was added to the treatment regimen, along with plasma exchange and molecular adsorbent recirculating system (MARS) therapy. Despite the given treatment, the clinical evolution led to a rapid deterioration, with multiple system organ failure (MSOF) and death on the 10th day after admission.

**Case 5:** A 58-year-old morbidly obese female, with a medical history characterized by diabetes mellitus, multiple cardiac pathologies (atrial fibrillation, left bundle branch block, NYHA group III cardiac failure, and hypertension), and oncological comorbidities (cervical cancer—operated on 6 months prior to admittance), along with a newly diagnosed chronic HBV infection, presented with progressive jaundice, firstly manifested during adjuvant radiotherapy. The patient was initially evaluated in a surgical unit, where an obstructive–mechanical cause of the jaundice was excluded and the patient was directed towards our hospital. Clinical evaluation revealed jaundice, multiple ecchymoses, and anasarca. Blood works showed mildly increased liver enzymes, hyperbilirubinemia (19.4 mg/dL), hypocholesterolemia, and a serology consistent with a recent HBV and HEV infection, possibly a superinfection (positive HBsAg, IgG HBcAb, HBeAb, and IgM HVEAb). Antiviral treatment with Entecavir was initiated along with corticotherapy and i.v. diuretics, with slow resolution of jaundice and a positive outcome overall.

The outcome was also marked by a central venous line bacterial infection and an admission to the ICU. Given the severity of heart failure, one cannot exclude an intricate cause of the liver damage and subsequent jaundice (heart failure combined with double hepatotropic infection).

## 3. Materials and Methods

In addition to the cases described in this article, we searched the medical literature for research papers regarding HEV and HBV infection over a period of 10 years (2014–2024). The search was performed using the PubMed database using certain keywords—(hev) AND (hbv)) AND (superinfection)) AND (chronic)) AND (acute)) AND (outcome)—and yielded nine relevant articles (seven retrospective and two prospective studies). It is important to note here that most of the medical literature focuses on the effect of acute hepatits on patients with underlying liver cirrhosis (LC).

## 4. Discussions

In the case of our described patients, the clinical outcome was mostly favorable, with one death (corresponding to 20%). It is hard to draw a conclusion, in this case, that the severe and fulminant nature of this infection was, in part, because of the dual infection. A dual pathogenic mechanism can be postulated: a direct, cytopathogenic liver injury (due to the HEV) and an immune-mediated liver injury (due to the chronic HBV infection).

Some [7] studies have shown that anti-HBV treatment does not influence the clinical outcome of the superinfection, while others have pointed towards a possible benefit [8]. From our case series, two patients received anti-HBV treatment, with a very favorable outcome, in spite of the severe comorbidities and a negative outcome. More studies are probably needed to ascertain a benefit from initiating anti-HBV treatment and to establish clinical and paraclinical criteria for treatment initiation. Other studies have shown that HEV-ACLF (acute-on-chronic-liver-failure) has the best survival rates, especially when compared to alcohol- or cryptogenic-induced ACLF [9,10], while also pointing out the fact that organ failure is a very important prognostic predictor, especially if present at an early stage of the infection. Case 4, with a fulminant course of the infection, with early onset of organ failure does not seem to contradict said findings.

The consensus in the limited medical literature available is that acute HEV infection superimposed on a pre-existing chronic HBV infection is associated with a more severe prognosis and is characterized by an acceleration of liver disease, especially in the case of patients already diagnosed with cirrhosis [11,12,13,14]. Excluding the first case (acute infection due to both VHE and VHB), the other four cases were diagnosed as an acute VHE infection on chronic VHB hepatitis. None of the four patients fulfilled the clinical, biological, or ultrasound criteria for advanced stage liver disease. Besides the fourth case, which had an unfavorable resolution, the other four patients had a favorable outcome, albeit with a slow normalization of liver tests. In one case, a relatively fast HB seroconversion was even observed.

Finally, when compared with HAV (hepatitis A virus), HEV superinfection in patients with an underlying HVB infection has a lower mortality and a better prognosis [15], highlighting the severity of a dual hepatotropic viral infection.

The main challenge in treating severe patients with this type of dual infection is determining which virus is responsible for the fulminant nature of the progression, if not both. Another thing that might need to be taken into consideration is the possible chronic HBV exacerbation in the context of the HEV-mediated immunosuppression.

Interestingly, one patient had a serology consistent with acute HBV and HEV hepatitis. Even with the dual infection, the clinical outcome was favorable.

A disadvantage of our study could be the fact that not all of the patients had their VHB viral loads determined. Determining the viral load in the setting of acute, maybe even fulminant hepatitis may be important in order to more precisely determine the amount of virus present in the organism and to quantify therapy response. Another limitation of this study is the absence of VHE molecular detection techniques in all of the cases presented. Cross-reactivity with other viral antigens has been demonstrated in a number of cases [16,17]. Especially in the case of immunocompromised patients, molecular testing, with its greater positive predictive [16] value, should be performed, complementary to serological testing.

We also mention the lack of medical studies available from resource-rich settings (countries with a high and very high HDI (Human Development Index)), which might be because of a relatively lower incidence of HEV infection and a lower prevalence of chronic HBV infection. Most of the articles found were limited to East, South, and Southeast Asia. On the other hand, on the European continent, there is no significant research tackling this sort of dual infection.

## 5. Conclusions

HEV acute infection superimposed on a chronic HBV infection is a relatively rare occurrence, especially in more developed countries. Given the increase in incidence of HEV infection, such cases might become more frequent.

In our case series, the clinical outcomes were mostly favorable, with one death (representing 20%). Only two patients were treated with anti-HBV medication (representing 40%), with one of them achieving HB seroconversion in a relatively short timeframe of 4 months. More studies are needed in order to describe the possible benefits of initiating HBV aethiological treatment in the case of HEV-HBV superinfection.

It is challenging to determine which virus is the main causal agent of the hepatic injury and therefore this condition is difficult to treat, with no preexisting guidelines regarding the treatment of such a severe yet rare occurrence. The intricate and not yet fully understood ways in which these two viruses can cause hepatic injury require further study. Needless to say, other co-infections with other hepatotropic viruses are also possible (HAV, HCV, CMV, etc.). Given the fact that there is no available vaccine for HEV hepatitis outside of China, it might be beneficial to advise patients already diagnosed with chronic HBV infection to undertaken supplementary measures to prevent infection with HEV and other hepatotropic viruses (proper sanitation and alimentary hygiene, availability of clean drinking water, etc.). Also, the importance of anti-HBV vaccination in patients with severe comorbidities can never be stressed enough.

## Figures and Tables

**Table 1:** Viral serological markers and molecular testing results in each presented case.

Case Number	AgHBs	HBcAb IgG	HBeAg	HBeAb	VHEAb IgM	VHBViral Load	VHDAb IgG
1	+	+	−	+	+	Test not performed	−
2	+	+	−	+	+	38,700,000 IU/mL	+
3	+	+	+	−	+	Test not performed	−
4	+	+	−	+	+	20,000,000 IU/mL	−
5	+	+	−	+	+	Test not performed	−

+: positive serology; −: negative serology.

## Data Availability

Data available upon request from the authors.
